# Complete chloroplast genome sequence of *Peganum harmala* (Zygophyllaceae)

**DOI:** 10.1080/23802359.2021.1909441

**Published:** 2021-04-07

**Authors:** Pei-Pei Jiao, Wei Si, Wen-Rui Qu, Shan-He Zhang, Tian-Ge Yang, Zhi-Hua Wu

**Affiliations:** a Xinjiang Production & Construction Corps Key Laboratory of Protection and Utilization of Biological Resources in Tarim Basin, College of Life Science, Tarim University, Alar, PR China; b College of Life Science and Technology, Huazhong Agricultural University, Wuhan, PR China; c Security Department, Tarim University, Alar, PR China; d Hubei Provincial Key Laboratory for Protection and Application of Special Plant Germplasm in Wuling Area of China, College of Life Sciences, South-Central University for Nationalities, Wuhan, PR China

**Keywords:** *Peganum harmala*, chloroplast genome, evolution

## Abstract

*Peganum harmala* L. is a perennial herbaceous plant belonging to the family of Zygophyllaceae, and is grows in semi-arid climates, such as Xinjiang, Gansu, Ningxia, Qinghai, and Inner Mongolia in China, and also Middle East and North Africa. This species is of high medicinal value. The complete chloroplast genome was reported in this study. The chloroplast genome with a total size of 159,957 bp consists of two inverted repeats (IR, 26,550 bp) separated by a large single-copy region (LSC, 88,098 bp) and a small single-copy region (SSC, 18,759 bp). Further annotation revealed the chloroplast genome contains 113 genes, including 79 protein-coding genes, 30 *tRNA* genes, and four *rRNA* genes. A total of 90 simple sequence repeats (SSRs) were identified in the chloroplast genome. This information will be useful for study on the evolution and genetic diversity of *Peganum harmala* in the future.

*Peganum harmala* L. is a perennial herbaceous plant belonging to the family of Zygophyllaceae, is grows in semi-arid climates, such as Xinjiang, Gansu, Ningxia, Qinghai, and Inner Mongolia in China and also Middle East and North Africa (Mahmoudian et al. 2002; Yonezawa et al. 2011). It is widely and frequently used as traditional folk medicine in Northwest and Northeast China, and also in Iranian (Niroumand et al. 2015). The alkaloids extracted from *P. harmala* seeds or whole herb have many pharmacological activities including insecticidal (Jbilou and Sayah 2007), antibacterial (Sodaeizadeh et al. 2010; Shaheen and Issa 2020), antihyperglycemic (Waki et al. 2007; Wang et al. 2015), antipsoriatic (Zeng et al. 2013), antitumor, and antileukemic effects (Zaker et al. 2007; Xue et al. 2015; Liu et al. 2012). Some of the pharmacologic properties have been confirmed by different studies in modern phytotherapy, harmine for example, was reported to show an unique therapeutic promise for human diabetes and anticancer therapy (Wang et al. 2015; Ding et al. 2019; Zhang et al. 2020). However, *P. harmala* harvested from Xinjiang region shows a higher genuineness of Chinese herbs for its significant higher harmine and harmaline contents (Sun et al. 2017). Due to the characteristics of uniparentally inheritance, highly conserved gene content and quadripartite organization, the chloroplast genome is widely used for used for research on plant phylogeny (Wu et al. 2014). In this study, to obtain the new insight into the phylogeny of *P. harmala*, we sequenced, assembled, and annotated the accurate chloroplast genome.

The materials of *P. harmala* in this study were collected from Aheqi County, Kizilsu Kirghiz Autonomous Prefecture, Xinjiang province of China (78°39.366′E, 40°57.837′N, 1876 m above sea level). The voucher specimen (TD-00562, *Peganum harmala* L.) was stored in the Germplasm Bank of Wild Species (http://www.genobank.org) and the herbarium of Tarim University. The complete genomic DNA was extracted using CTAB method (Doyle JJ and Doyle JL 1987) and sequenced using the Illumina NovaSeq 6000 platform at Majorbio Company (Shanghai, China). The assembly of chloroplast genome was described as follows in brief. First, the clean reads were quality-controlled by FastQC version 0.11.9 (http://www.bioinformatics.babraham.ac.uk/projects/fastqc/). Then, the whole chloroplast genome was assembled using GetOrganelle version 1.7.3 (Jin et al. 2020). Finally, to check the accuracy of final assembly, the slimmed assembly graph and selected target assembly graph were further visualized by Bandage version 0.8.1 (Wick et al. 2015). Gene annotation was performed using CPGAVAS2 (http://47.96.249.172:16019/analyzer/annotate) (Shi et al. 2019), and PGA (https://github.com/quxiaojian/PGA) (Qu et al. 2019). Annotations of protein-coding sequences were confirmed using BLASTx in NCBI. The complete chloroplast genome was 159,957 bp (MW307830) and composed of two IRs of 26,550 bp each, which divide into a large single copy (LSC) region of 88,098 bp and a small single copy (SSC) region of 18,759 bp, the average GC content was 37.53%. The chloroplast genomes encoded 113 functional genes, including 79 protein-coding genes, 30 *tRNA* genes, and four *rRNA* genes. A total of 90 SSR markers ranging from mononucleotide to hexa-nucleotide repeat motif were identified in *P. harmala* chloroplast genome.

To explore the phylogenetic relationship of *P. harmala* within Zygophyllaceae, additional 11 species from Sapindales and six species from Zygophyllaceae were studied. With the *Malva parviflora* and *Malva wigandii* as the outgroups, the phylogenetic trees were built from the 79 protein-coding gene by maximum-likelihood (ML) and Bayesian inference (BI) (Figure 1). The ML tree was generated using IQ-TREE version 2.1.2 (Nguyen et al. 2015) based on the best model of TVM + F+R3 and 1000 bootstrap replicates, and BI analysis was performed in MrBayes version 3.2.7 (Ronquist et al. 2012). This result showed that the analyzed *P. harmala* was closer to the species of *Nitraria tangutorum* and *Nitraria roborowskii.*

**Figure 1.**
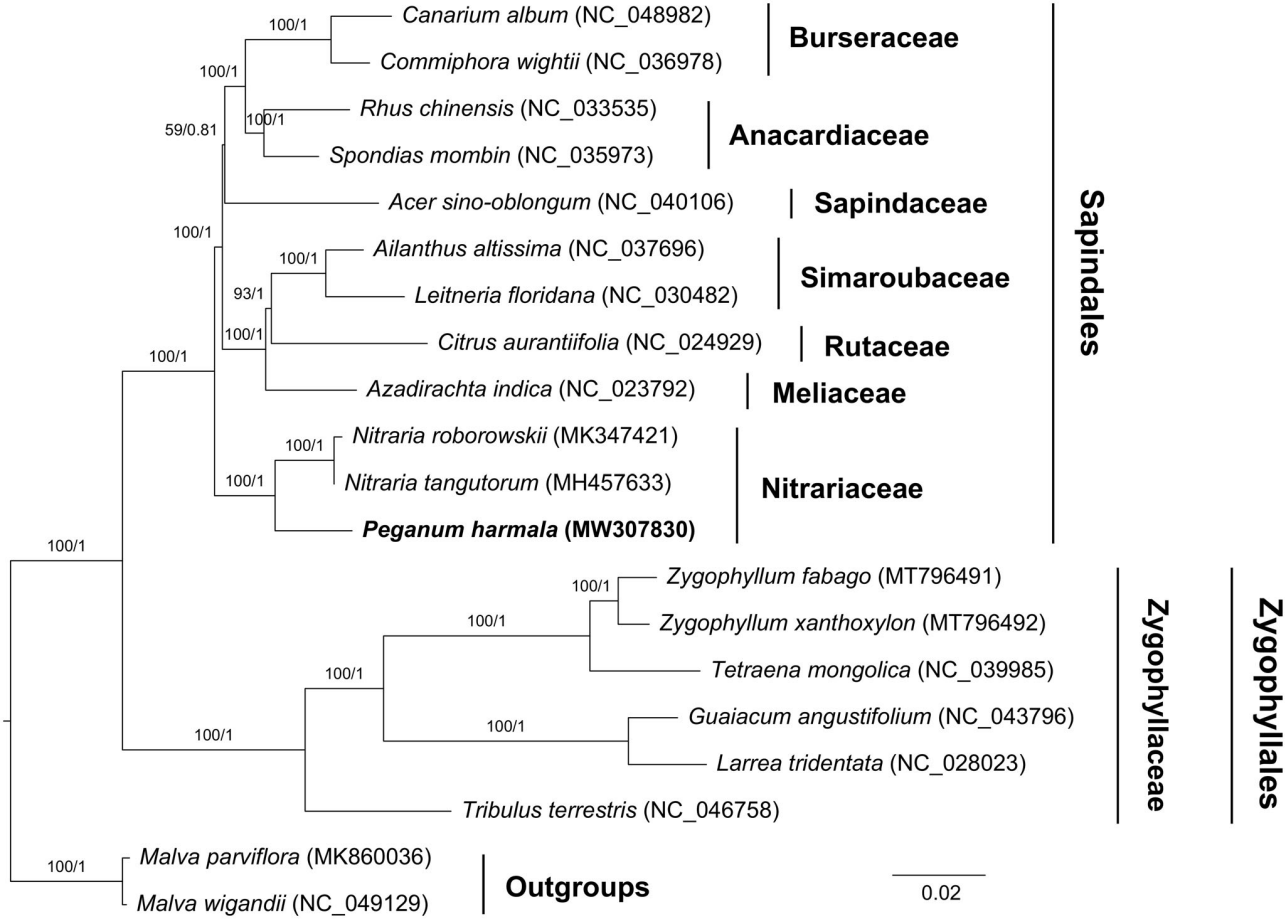
Phylogenetic tree reconstructed by maximum-likelihood (ML) and Bayesian inference (BI) analysis based on the 79 chloroplast protein-coding genes from 20 species.

## Data Availability

The genome sequence data that support the findings of this study are openly available in GenBank of NCBI at [https://www.ncbi.nlm.nih.gov] (https://www.ncbi.nlm.nih.gov/) under the accession no. MW307830. The associated ‘BioProject’, ‘SRA’, and ‘Bio-Sample’ numbers are PRJNA692776, SRR13451271, and SAMN17369979, respectively.
